# Isolation of salivary cell-free DNA for cancer detection

**DOI:** 10.1371/journal.pone.0285214

**Published:** 2023-05-02

**Authors:** Patricia J. Brooks, Ethan Z. Malkin, Steven De Michino, Scott V. Bratman

**Affiliations:** 1 Princess Margaret Cancer Centre, University Health Network, Toronto, ON, Canada; 2 Department of Medical Biophysics, Temerty Faculty of Medicine, University of Toronto, Toronto, ON, Canada; 3 Department of Radiation Oncology, Temerty Faculty of Medicine, University of Toronto, Toronto, ON, Canada; Nelson Mandela African Institute of Science and Technology, UNITED REPUBLIC OF TANZANIA

## Abstract

Saliva is an emerging source of disease biomarkers, particularly for cancers of the head and neck. Although analysis of cell-free DNA (cfDNA) in saliva holds promise as a liquid biopsy for cancer detection, currently there are no standardized methodologies for the collection and isolation of saliva for the purposes of studying DNA. Here, we evaluated various saliva collection receptacles and DNA purification techniques, comparing DNA quantity, fragment size, source, and stability. Then, using our optimized techniques, we tested the ability to detect human papillomavirus (HPV) DNA– a bona fide cancer biomarker in a subset of head and neck cancers– from patient saliva samples. For saliva collection, we found that the Oragene OG-600 receptacle yielded the highest concentration of total salivary DNA as well as short fragments <300 bp corresponding to mononucleosomal cell-free DNA. Moreover, these short fragments were stabilized beyond 48 hours after collection in contrast to other saliva collection receptacles. For DNA purification from saliva, the QIAamp Circulating Nucleic Acid kit yielded the highest concentration of mononucleosome-sized DNA fragments. Freeze-thaw of saliva samples did not affect DNA yield or fragment size distribution. Salivary DNA isolated from the OG-600 receptacle was found to be composed of both single and double-stranded DNA, including mitochondrial and microbial sources. While levels of nuclear DNA were consistent over time, levels of mitochondrial and microbial DNA were more variable and increased 48 hours after collection. Finally, we found that HPV DNA was stable in OG-600 receptacles, was reliably detected within the saliva of patients with HPV-positive head and neck cancer, and was abundant among mononucleosome-sized cell-free DNA fragments. Our studies have defined optimal techniques for isolating DNA from saliva that will contribute to future applications in liquid biopsy-based cancer detection.

## Introduction

Head and neck cancers, of which head and neck squamous cell carcinomas (HNSCCs) make up the majority, are the 7^th^ most common cancer worldwide. Generally, HNSCCs are associated with a poor 5-year overall survival of less than 50%, a number that has remained unchanged for many years due to diagnoses being made most frequently at locoregionally advanced disease stage [1]. Diagnosis is dependent on tissue biopsies, which are invasive, site-specific and technique-sensitive, where sampling errors may account for up to 60% of inaccurate diagnoses and fail to account for field cancerization [2]. Therefore, techniques for earlier detection that provide insight at the malignancy status of the entire oral cavity are of utmost importance to improve both patient morbidity and mortality. Additionally, DNA from more distant cancer types have been detected within saliva, furthering the need for standardized collection and isolation techniques [3].

The use of bodily fluids to conduct liquid biopsies is a promising approach for non-invasive cancer detection and diagnosis of disease, prognostication, assessment of response to therapy, and identifying potential molecular targets. Circulating cell-free DNA (cfDNA) can be found in liquid biopsies and arises from multiple mechanisms including apoptosis, necrosis and active secretion [4]. Fragments of cfDNA can be of varying length, ranging from ~140–170 base pairs (bp), or multiples thereof, which correspond to mononucleosomes [5]. Circulating tumor DNA (ctDNA) is a fraction of this DNA that arises from tumoral cells and comprises a group of biomarkers that hold the potential for replacing tissue biopsy. Analysis of these fragments within cfDNA provides a window into cancer-specific genetic and epigenetic aberrations [6].

While peripheral blood plasma-derived liquid biopsies dominate the literature, saliva is another promising source of biomarkers in HNSCCs due to proximity to the primary disease as well as ease of collection. Proof-of-principle studies have found recurrent mutations and aberrant DNA methylation from primary HNSCC tumours within both plasma and saliva [7–9]. In early stages of disease, especially those cancers in close proximity to the oral cavity, tumour-derived DNA may be more readily detectable in saliva compared with peripheral blood plasma [7], and the combination of both saliva and plasma analysis may improve detection accuracy [10].

Currently, there are no standardized protocols for the collection and processing of saliva for obtaining DNA-derived biomarkers. Some studies analyzing salivary DNA do not differentiate from total cellular versus cfDNA or the benefits of each as biomarkers. To further complicate this, contributions to salivary cfDNA can involve healthy and tumoral DNA fragments, mitochondrial DNA (mtDNA), exosomal DNA, and microbial DNA from the oral flora. Here we present the conditions for isolation and processing of salivary DNA for use in downstream biomarker detection. Additionally, we analysed the contributions of each DNA type in salivary total DNA and performed a proof-of-principle study of human papillomavirus (HPV) detection in salivary DNA of HPV-positive oropharyngeal cancer patients. Our findings can help to advance a standardized technique for widespread use of salivary-based DNA isolation and processing to improve cancer detection.

## Materials & methods

### Sample collection

All work was approved by the Research Ethics Board at University Health Network (21–5592.0). Written informed consent was obtained from all participants for inclusion in the study. Samples were collected from healthy donors and from patients with newly diagnosed HPV-positive oropharyngeal carcinoma as confirmed by tissue biopsy with >70% p16-positivity by immunohistochemistry (see Table 1 for patient demographics and staging information). Saliva was passively collected by subjects expectorating into three different collecting tubes Oragene OG-600 (DNA Genotek, #OG-600), Norgen cfDNA 5cc (Norgen Biotek, #18111), and 15 mL DNAse/RNAse-free conical tubes (FroggaBio, #TB15-25). All samples from healthy donors were collected at the same time of day with abstinence from eating and drinking for 30 minutes prior to donation. Saliva samples were processed after the allotted incubation times (0, 2, 12, 24, 48, 72, or 168 hours), or frozen at -80°C for at least 2 weeks to ensure homogeneity. 10 mL of blood was collected in cell-free DNA blood collection tubes (Streck, Cell-Free DNA BCT®, #230471). Plasma was isolated from the blood within 48 hours following the manufacturer’s instructions. High-risk HPV DNA for the spike-in experiments was generated by purifying genomic DNA from the HPV16-positive SiHa cell line (ATCC #HTB-35) that contains a single integrated copy. This DNA was subsequently fragmented to a median length of approximately 200 bp by sonication (Covaris M220) as confirmed by BioAnalyzer analysis. The sheared cell line DNA containing HPV16 DNA was utilized in the spike-in experiments by adding 2 μL of 1.25ng/μL per mL of saliva immediately after saliva collection from healthy donors.

**Table 1:** HPV16-positive OPSCC patients.

Patient ID	Age	Sex	Ethnicity	Primary Site	TNM	AJCC 8th Ed. Stage	Plasma HPV DNA (copies/mL)	Saliva HPV DNA (copies/mL)
OPSCC 1	48	M	Caucasian	Base of Tongue	T2N3M0	III	2440	200
OPSCC 2	67	M	Caucasian	Base of Tongue	T2N2M0	II	40	202
OPSCC 3	55	F	Caucasian	Palatine Tonsil	T1N1M0	I	0	8833

### DNA purification, quantification, and size selection

DNA purification from saliva samples was performed utilizing three different approaches. First, cellular debris was removed from the samples by centrifuging at 300g for 20 minutes followed by 10,000g for 20 minutes. Next, the supernatant was used for DNA purification using either ethanol precipitation (DNAGenotek, prepIT #PT-L2P-5), Dneasy Blood & Tissue (Qiagen #69504), or QIAamp Circulating Nucleic Acid (Qiagen #55114) kits following the manufacturers’ protocols. The total quantities of double-stranded DNA (dsDNA) and single-stranded DNA (ssDNA) were measured by Qubit fluorometry (Invitrogen). DNA fragment sizing and quantification was performed using the 2100 Bioanalyzer (Agilent Technologies, High Sensitivity DNA Chips) capillary electrophoresis system. AMPure XP magnetic beads (Beckman Coulter) were used to isolate DNA fragment sizes of >300 bp and <300 bp for analysis according to the manufacturer’s instructions; a 50 bp DNA ladder (New England Biolabs, #N3236L) was used for confirmation of isolated fragment size.

### DNA quantification by qPCR and ddPCR

Nuclear DNA was quantified by qPCR using a Bio-Rad CFX96 Touch Real-Time PCR detection system targeting the second open reading frame of *LINE1*, a retrotransposon sequence with approximately 100,000 repetitive elements dispersed throughout the genome. *LINE1* has an extremely high copy number that allows for highly sensitive detection of nuclear DNA [11]. The following primer sequences were used: 5’-TCTGCCTTCATTTCGTTATGTACC-3’ (forward); 5’-TCACTCAAAGCCGCTCAACTAC-3’ (reverse). Primers were obtained from Integrated DNA Technologies. Standard curves were generated using known concentrations of Human Male Genomic DNA (Promega). PCR conditions were as follows: DNA polymerase activation at 95°C for 3min, followed by 40 cycles of denaturation at 95°C for 10s and annealing/extension at 55°C for 30s. The sample volume for each reaction was 10μL, and a melt curve was included with each run to ensure a single peak with no off-target amplification. A DNA-free negative control was included with each run to ensure that all samples were above the minimum threshold of detection for this assay.

Mitochondrial and microbial DNA, as well as HPV DNA, were quantified by ddPCR using the QX200 Droplet Digital PCR System (Bio-Rad). Mitochondrial and microbial DNA were quantified in a duplex assay. Mitochondrial DNA was quantified by targeting the mitochondrial protein-coding gene NADH-ubiquinone oxidoreductase chain 1 (*MTND1*) as described elsewhere [12] using the following sequences: 5’-CCCTAAAACCCGCCACATCT-3’ (forward); 5’-GAGCGATGGTGAGAGCTAAGGT-3’ (reverse); 5’-FAM-CCATCACCCTCTACATCACCGCCC-3’ (probe). Microbial DNA was quantified by targeting a highly conserved region of the microbial 16s rRNA gene. Primers for microbial DNA were modified from those described elsewhere [13] and the sequences are as follows: 5’- AACAGGATTAGATACCCTGGTAG-3’ (forward); 5’-GGTTCTKCGCGTTGCWTC-3’ (reverse, where K = G or T and W = A or T); 5’-FAM-AGCGGTGGAGCATGTGG-3’ (probe). HPV DNA was quantified by targeting both HPV16 E6 (primers: 5’- ACTGTGTCCTGAAGAAAAGCA-3’ (forward); 5’-GTCCACCGACCCCTTATATT-3’ (reverse); 5’-FAM-ACATCTGGACAAAAAGCAAAGATTCCA-3’ (probe)) and HPV E7 (primers: 5’-GAGGAGGATGAAATAGATGGTC-3’ (forward); 5’-GAGCGATGGTGAGAGCTAAGGT-3’0 (reverse); 5’-GAGCGATGGTGAGAGCTAAGGT-3’1 (probe)) in a duplex assay and averaging the copy number for each sample [14]. All primers and primer/probe sets were obtained from Integrated DNA Technologies.

Mitochondrial DNA (mtDNA) primers were validated by qPCR on purified human mtDNA isolated from the human colon cancer cell line HCT116 (ATCC #CCL-247) using a mtDNA isolation kit (Abcam), as per the manufacturer’s instructions. Microbial DNA primers were validated by qPCR on a community microbial DNA standard (Zymo Research) containing DNA from *E*. *coli*, *B*. *subtilis*, *E*. *faecalis*, *L*. *fermentum*, *L*. *monocytogenes*, *P*. *aeruginosa*, *S*. *enterica*, and *S*. *aureus*. HPV DNA primers were validated by qPCR on purified genomic DNA from the HPV-positive squamous cell carcinoma cell line, SiHa. Next, an 8-step ddPCR temperature gradient was conducted to determine optimal amplification conditions (58°C for mitochondrial/microbial DNA assay, 56°C for HPV DNA assay). Subsequently, purified DNA samples were run on ddPCR according to the manufacturer’s instructions. A standard containing relevant template DNA was included in each run, and the signal threshold was set according to this standard. A blank was also included in each run to preclude sample contamination. Only samples with >12,000 events were included in analyses, as per Bio-Rad’s suggested protocols. Data was analyzed using QuantaSoft Analysis Software (Version 1.7.4.0917; Bio-Rad).

### Statistical analysis

All experiments were done in triplicate and expressed as mean +/- the standard deviation. Student t-tests or one-way ANOVA with Tukey’s post-hoc tests performed (GraphPad Prism v9.0.1).

## Results

### Mononucleosome-sized DNA fragments are present in saliva and preserved by the Oragene collection tube

Short mononucleosome-sized DNA fragments within cfDNA have emerged as important substrates for liquid biopsy biomarker tests. The quantity and stability of these fragments within saliva have not been carefully studied following different salivary DNA isolation techniques. We first evaluated total and mononucleosome-sized DNA content in saliva from a healthy donor using three different collection receptacles: Oragene OG-600, Norgen cfDNA 5mL, and empty 15 mL DNAse/RNAse-free conical tubes (Neat). Whereas the Norgen and Neat collection techniques are intended to isolate only cfDNA, the OG-600 collection tube contains lysis buffer and, therefore, isolates both cfDNA and cellular DNA [15]. A flowchart depicting the analyses performed on the different sample types has been included (S1 Fig).

**Fig 1:**
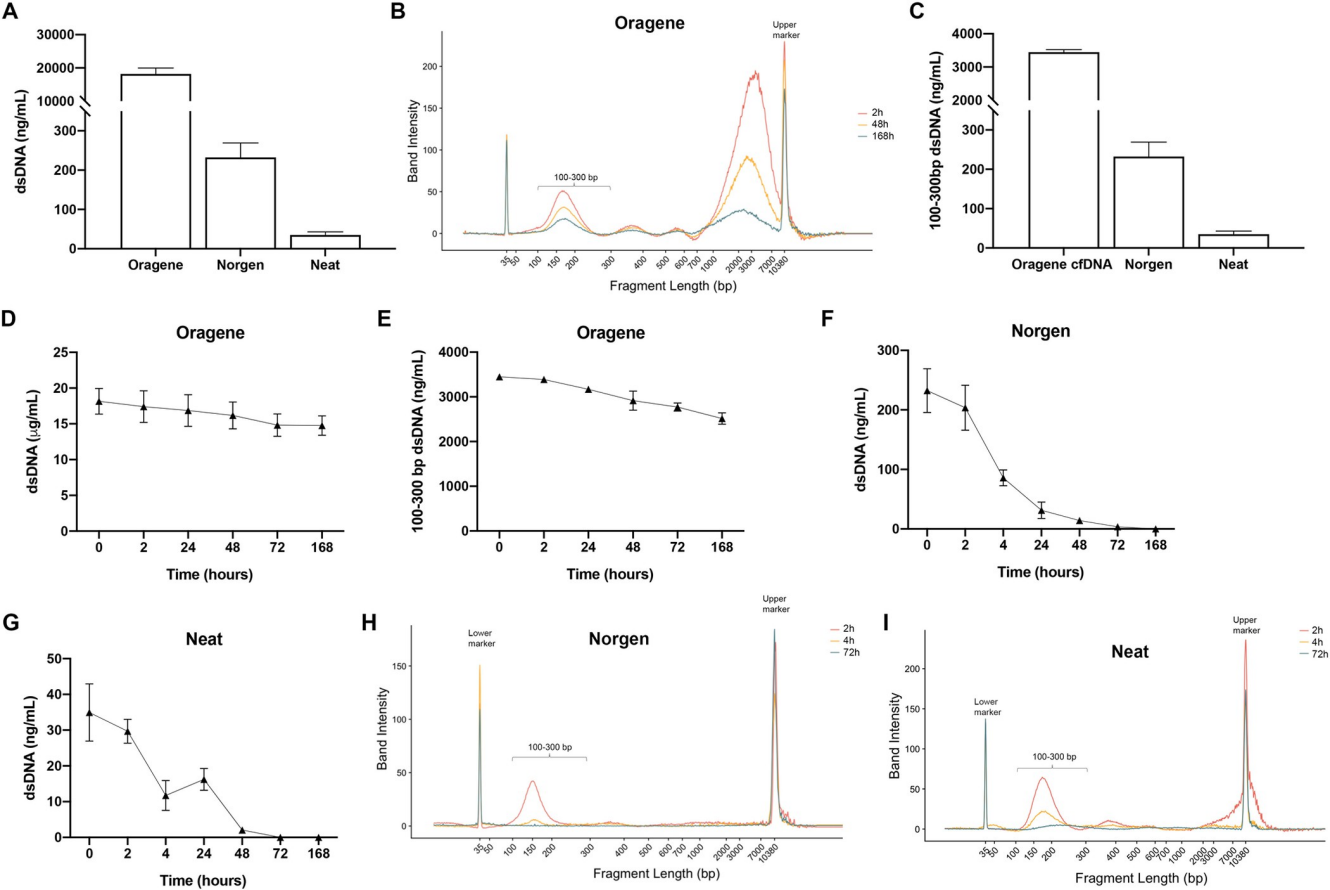
Oragene OG-600 collection tubes yield the most DNA from donor salivary DNA at all time points. Quantification of dsDNA utilizing three collection tubes for Donor A; Oragene OG-600, Norgen 5 mL, and 15 mL DNAse/RNAse-free conical tubes (Neat) followed by DNA isolation with the QIAamp Circulating Nucleic Kit after incubation at room temperature (mean ± standard deviation). A) Total DNA content per milliliter of saliva at time zero for each collection tube as determined with dsDNA Qubit fluorometry and B) Electropherogram of QIAamp Circulating Nucleic Acid Kit isolation from OG-600 at 2, 48 and 168 hours after collection. C) DNA content per milliliter of saliva in the 100–300 bp range only from the OG-600 tube. D) DNA content per milliliter of saliva with OG-600 collection tubes as determined with dsDNA Qubit fluorometry at varying intervals for total DNA and E) 100–300 bp as determined using DNA electrophoresis after incubation. F) DNA content from at varying intervals after collection with Norgen and G) Neat tubes. H) Electropherogram of DNA after QIAamp Circulating Nucleic Acid Kit isolation 2, 4 and 72 hours after collection from Norgen tubes and I) Neat tubes. All data points represent n = 3.

Compared with Neat saliva collection, both Norgen and Oragene tubes yielded consistently higher concentrations of DNA (Fig 1A). As expected, DNA yields were dramatically higher using Oragene tubes due to the presence of cellular DNA. Gel electrophoresis analysis revealed a preponderance of long DNA fragments >1 kb, consistent with cellular DNA released through cell lysis (Fig 1B). Despite these long fragments representing the majority of DNA content isolated from Oragene tubes, there was also distinct presence of DNA fragments 100 to 300 bp in size, corresponding to mononucleosome-sized DNA fragments (Fig 1C). Quantification of only these fragments revealed that their abundance remained higher using Oragene tubes than either Norgen tubes or Neat saliva.

Salivary DNA has been reported to be stable to degradation over time when collected with Oragene tubes [16], but the stability of mononucleosome-sized DNA fragments is not yet known. We therefore tested DNA quantity and size distribution following sample incubation at room temperature for varying intervals up to one week (168 hours). Notably, there was minimal change in both total (Fig 1D; 5.7% loss per day) and mononucleosome-sized DNA fragments (Fig 1E; 1.8% loss per day) over these time intervals. In contrast, when saliva was collected with either Norgen tubes or Neat, both total and mononucleosome-size DNA fragments rapidly degraded within 48 hours (Fig 1F–1I).

### The QIAamp circulating nucleic acid kit yielded the most consistent amount of salivary DNA after Oragene tube incubation

**Fig 2:**
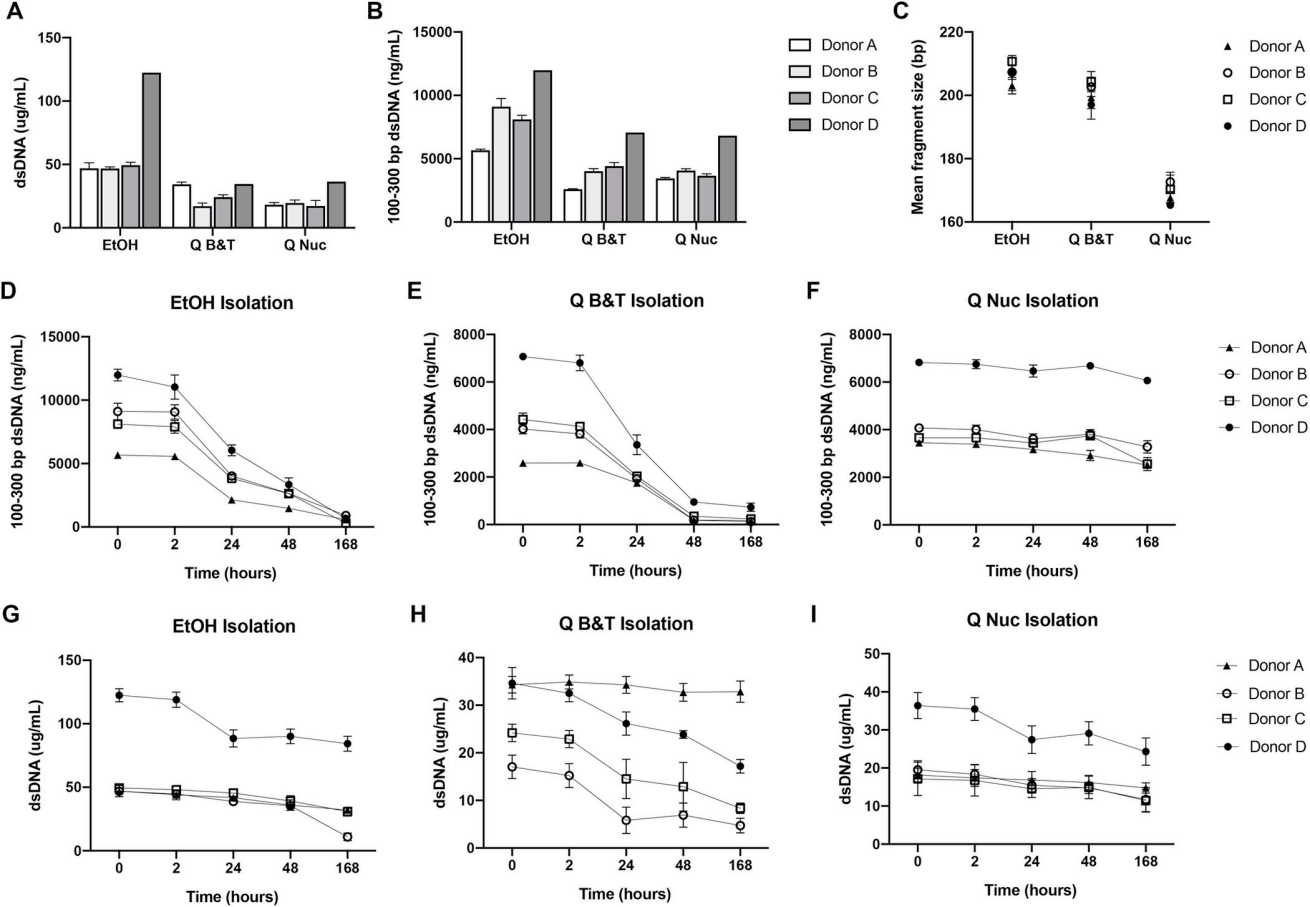
Ethanol isolation yields the highest amount of total DNA when utilizing the OG-600 tubes, however the QIAamp circulating nucleic acid kit produces the most consistent yield of the cfDNA-sized fragments. DNA isolation from Oragene OG-600 collection tubes in four donors utilizing three different DNA isolation techniques; column-free ethanol (EtOH) precipitation, DNeasy Blood & Tissue (Q B&T), and QIAamp Circulating Nucleic Kits (Q Nuc) after incubation at room temperature (mean ± standard deviation). A) Total dsDNA content per milliliter of saliva and B) 100–300 bp DNA at time zero after each DNA isolation technique as determined with dsDNA Qubit fluorimetry. C) Mean fragment size in the 100–300 bp range of DNA isolated by each isolation technique. D-F) Concentration of dsDNA within the 100–300 bp range at varying intervals as determined using DNA electrophoresis and G-I) total DNA. All data points represent n = 3.

Having determined that Oragene tubes produced the highest abundance of total and mononucleosome-sized DNA, we next asked whether the DNA purification technique used following saliva collection had an impact on DNA yield and stability. We collected saliva from healthy donors and tested three different techniques for DNA purification from the saliva: ethanol precipitation, the Qiagen Dneasy^®^ Blood & Tissue kit, and the QIAamp^®^ Circulating Nucleic Acid kit that was used in experiments presented in Fig 1. At time zero, ethanol precipitation gave the highest yield of both total and mononucleosome-sized DNA (Fig 2A and 2B). However, the mean fragment size of the mononucleosome-sized DNA across the donors was higher when purified by ethanol precipitation (207 ± 4 bp) or the Dneasy kit (201 ± 4 bp) as opposed to the QIAamp Circulating Nucleic Acid kit (169 ± 4 bp), suggesting that the first two former methods may be inefficient at purifying short DNA fragments from saliva.

Furthermore, following room temperature incubation of Oragene tubes for up to 168 hours there was significant degradation of the shorter mononucleosome-sized DNA fragments when purified by either ethanol precipitation (Fig 2D; 92.6 ± 3.0% loss after 168 hours) or by the Dneasy kit (Fig 2E; 93.6 ± 2.9% loss after 168 hours, p = 0.003). In contrast, the majority of these fragments were preserved when purified by the QIAamp Circulating Nucleic Acid kit (Fig 2F; 21.9 ± 8.6% loss after 168 hours, *p* = 0.0005 and 0.0003, respectively). This dramatic difference in stability of the short DNA fragments was not observed when considering total DNA content irrespective of fragment size (Fig 2G–2I) (31.2 ± 11.5% loss after 168 hours for QIAamp Circulating Nucleic Acid kit versus 44.3 ± 20.4 for ethanol precipitation (*p* = 0.220) and 47.7 ± 28.4 for Dneasy kit (*p* = 0.226)).

### DNA yield and fragment length of saliva samples is unaltered with freezing preservation techniques

**Fig 3:**
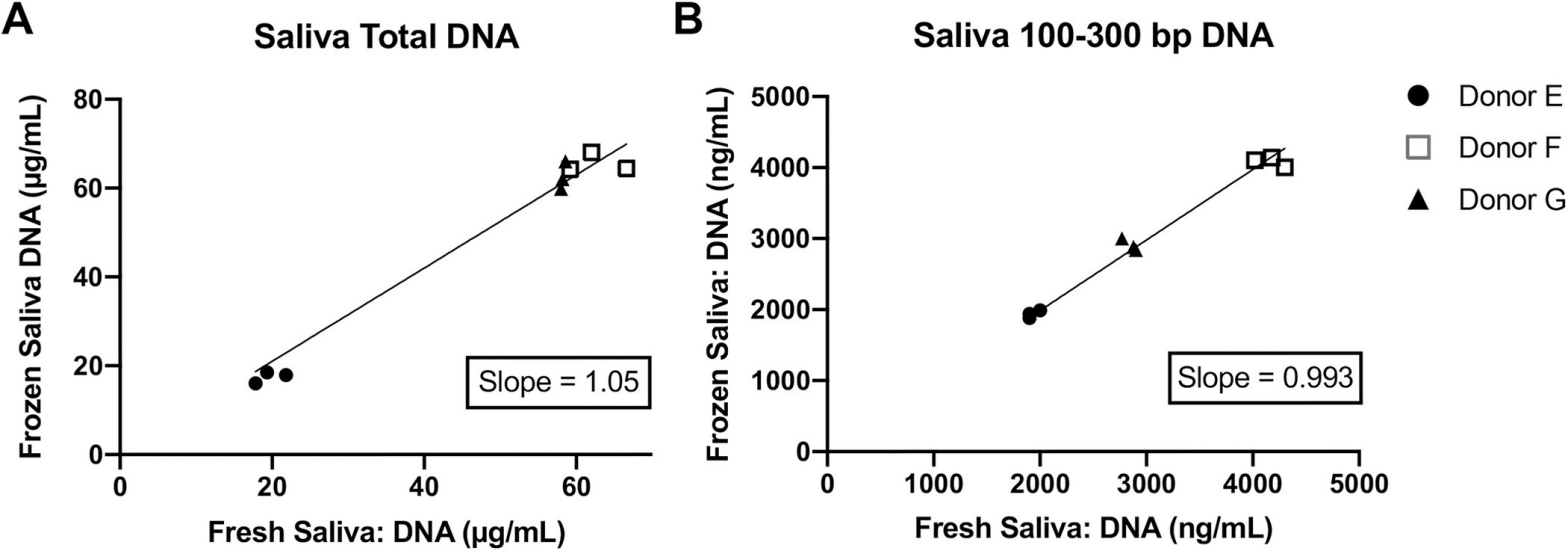
Total and cfDNA quantification remains unchanged with freezing. Linear regression of frozen versus fresh saliva-obtained DNA using OG-600 collection tubes in three healthy donors with immediate DNA isolation or freezing at -80°C followed by quantification using the QIAamp Circulating Nucleic Acid kit. A) Total DNA concentration as determined with dsDNA Qubit fluorometry and B) 100–300 bp fragment concentrations determined using DNA electrophoresis.

We next asked whether freezing of saliva prior to DNA isolation would impact DNA yield or fragment length distribution. Saliva from three healthy donors was collected using Oragene tubes, and DNA was purified with the QIAamp Circulating Nucleic Acid kit either immediately following collection or after storage at -80°C. Concentration of total salivary DNA (Fig 3A) and that of mononucleosome-sized fragments (Fig 3B) were highly correlated among the donors with no evidence of degradation. Moreover, freeze-thaw did not cause a significant difference in the mean fragment size of the mononucleosome-sized DNA across the donors (paired t-test; *p* = 0.4096). These results suggest that saliva collected in Oragene tubes can be stored at -80°C and subsequently used for DNA purification and downstream biomarker analyses.

### Salivary DNA sources include genomic, mitochondrial, and microbial DNA

**Fig 4:**
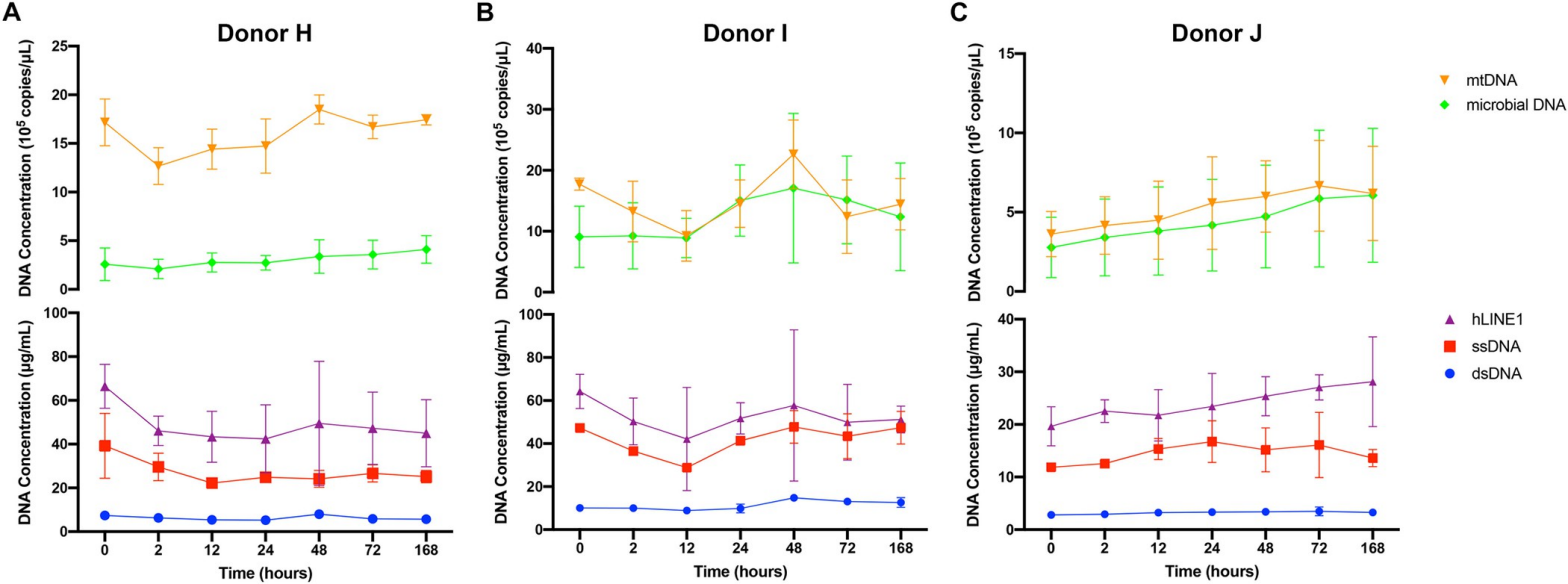
Salivary DNA derives from genomic, mitochondrial, and microbial sources. Quantification of source DNA from Oragene OG-600 collection tubes in three donors after incubation at room temperature and isolation with QIAamp Circulating Nucleic Acid kit at various time points (mean ± standard deviation). Total dsDNA and ssDNA as determined by Qubit fluorometry, hLINE1 from qPCR, and mtDNA and microbial DNA by droplet digital PCR (ddPCR). All data points represent n = 3.

In order to discover the source and types of DNA present within saliva, we quantified distinct DNA species using fluorometric (ssDNA and dsDNA) and PCR (human genomic DNA, mitochondrial DNA [mtDNA], and microbial DNA) techniques. Saliva from three healthy donors was collected in Oragene tubes, and following room temperature incubation for up to 168 hours, salivary DNA was purified using the QIAamp Circulating Nucleic Acid kit. Yields of ssDNA, dsDNA, and human genomic DNA were relatively consistent over the incubation course. Interestingly, both microbial DNA and mtDNA were present in all samples and increased during the incubation period for the 3 donors, particularly at the 48 hour timepoint (Fig 4). These results support processing of saliva collected in Oragene tubes in less than 48 hours to ensure consistent makeup of DNA constituents.

### High-risk HPV16 DNA is detected in fixed saliva among fragments smaller than 300 bp

**Fig 5:**
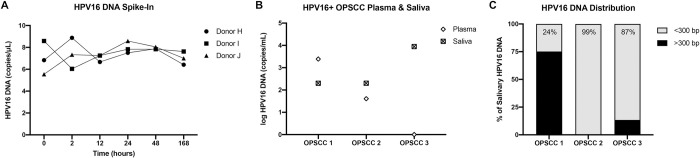
HPV DNA is detectable in saliva specimens using OG-600 tubes and QIAamp circulating nucleic acid isolation kit. A) HPV16 DNA was added to healthy donor saliva samples in OG-600 tubes at time zero, with DNA isolation at various time points and detection using droplet digital PCR (ddPCR). B) HPV16 DNA per mL of saliva or blood plasma from pretreatment OPSCC patients with HPV16+ OPSCC. DNA isolation occurred 24 hours after collection. C) HPV16 copy number as detected by ddPCR in Ampure-bead size-selected saliva specimens from HPV16+ OPSCC patients, with the percentage of contribution from DNA fragments <300 bp depicted.

We then asked whether our optimized saliva collection methods could be used for robust detection of tumour DNA. First, we collected saliva from healthy donors into Oragene tubes and spiked in high-risk HPV16 DNA. The tubes were incubated at room temperature for varying intervals up to 168 hours. At specific time points, we purified DNA with the QIAamp Circulating Nucleic Acid kit and conducted droplet digital PCR (ddPCR) analysis to quantify HPV16 DNA copy number. We observed no change in the HPV16 DNA copy number over time (Fig 5A), indicating stability within saliva in Oragene tubes. We expected 10 copies of HPV DNA per μL of saliva, and achieved relatively high yields, averaging 7.4 copies per μL of saliva, giving a yield of 74%. No HPV DNA was detected in any of the healthy control samples.

Next, we collected both saliva and peripheral blood plasma samples from newly diagnosed HPV-positive oropharyngeal squamous cell carcinoma (OPSCC) patients and analyzed the samples for HPV DNA abundance. HPV16 DNA was detected in 3/3 saliva samples (range: 200–8833 copies/mL of saliva) but in only 2/3 plasma samples (range: 0–2440 copies/mL of plasma) (Fig 5B). None of the healthy donor samples showed any detectable HPV DNA. Interestingly, the patient with HPV16 DNA detected in saliva but not plasma had earlier stage disease with more limited nodal metastasis than the two patients with HPV16 DNA detected in both saliva and plasma (Table 1).

Finally, we asked whether HPV16 DNA was present among the shorter mononucleosome-sized salivary DNA fragments that are preserved by Oragene tubes. We separated salivary DNA fragments into those greater than and less than 300 bp in length using bead-based size selection. All 3 saliva samples harbored HPV16 DNA among the fraction with smaller fragment lengths (Fig 5C). Moreover, in 2/3 saliva samples, DNA fragments smaller than 300 bp contained the vast majority of HPV16 DNA (99.4 and 86.6%, respectively).

## Discussion

As the known clinical utility of blood-based ctDNA testing expands, there is increasing interest in evaluating alternative biofluids as sources of ctDNA for complementary diagnostic applications. Saliva presents distinct advantages over blood particularly for localized HNSCC due to the proximity to primary tumors and ease of collection. Robust and generalizable studies of saliva for biomarker applications will require standardized protocols for salivary DNA isolation. Additionally, understanding the sources of DNA within saliva that could be utilized for biomarker development is of utmost importance. Here, we conducted the first comprehensive analysis of saliva collection and processing methods for the purposes of downstream salivary DNA purification and biomarker analysis.

We confirmed that the stability of DNA, and specifically mononucleosome-sized cfDNA, without nuclease-inactivating agents is poor. Saliva is composed of a number of enzymes, including nucleases responsible for DNA degradation [17]; therefore, rapid loss of DNA integrity in saliva is to be expected with increased incubation periods. Processing of samples either needs to occur rapidly upon collection or else with the aid of appropriate stabilizing reagents. Our conclusions are supported by other studies that have stipulated either immediate processing [3] or immediate cold storage [18] of saliva for cfDNA analysis. Another group that utilized the Oragene tube assessed the ability to recover total salivary DNA in various conditions but did not focus on cfDNA specifically [19].

Different techniques for DNA purification from saliva may also impact yield and stability. Although we observed higher salivary DNA yield with ethanol precipitation as opposed to spin column techniques, short DNA fragments were more efficiently purified and by far the most stable using the QIAamp Circulating Nucleic Acid kit. This observation is consistent with prior studies revealing superior recovery rates of spiked-in ladders of short DNA fragments using the QIAamp Circulating Nucleic Acid kit [20, 21], but it is the first time this has been demonstrated in salivary DNA.

We observed negligible effect of saliva freeze-thaw on DNA yields, especially those in the cfDNA range. The mean fragment length also remained unchanged after freeze-thaw when compared with immediate processing. These results indicate that mononucleosome-sized DNA fragments within saliva can be preserved for subsequent purification and batched analysis. Combined with other published studies showing stability of total salivary DNA following freeze-thaw or when stored at -80°C [22–24], our study lends support to biobanking efforts for future saliva-based liquid biopsy research.

Salivary DNA comes from multiple sources including the nucleus, mitochondria, and microbes. We found that there was an increase in microbial DNA and mtDNA in some samples within 48 hours of collection. It is possible that niduses of bacteria persisted in the tube allowing for microbial growth over the incubation period. It is understood that there will be a certain amount of unavoidable microbial DNA in saliva samples due to the presence of oral microbiome in both healthy and diseased states [17], but limiting the quantity could be important for the fidelity of certain downstream analyses such as next-generation sequencing [25, 26]. Mitochondrial DNA showed increases over time, indicating that the release of DNA from these organelles by the lysis buffer does not occur as quickly as the cellular genomic DNA. Moreover, a stable contribution from distinct pools of mtDNA might be important for liquid biopsy applications [27–29]. While it is possible that some contribution of non-genomic DNA may also arise from exosomes and other extracellular vesicles, due to the presence of lysis buffer the ability to delineate these sources by standard techniques is not possible [30]. Taken together, we recommend that saliva from Oragene tubes is either frozen at -80°C or subjected to DNA purification within approximately 24 hours of collection.

Detection of tumor DNA in saliva has many potential biomarker applications. We found that tumor-derived HPV DNA was detectable and stable in saliva collected in Oragene tubes. Moreover, in a proof-of-concept study, for some patients HPV DNA levels were higher in saliva than in plasma. Tumor DNA can reach the saliva either through direct shedding from a mucosal tumor or filtering from plasma within salivary glands [31]. This notion that saliva is a filtered version of blood plasma has been supported by observations that liquid biopsy genotyping of lung cancer is concordant between plasma and salivary DNA [32].

Our assessment of HPV-positive OPSCC patient pre-treatment saliva and plasma samples confirmed that our collection and isolation techniques were adept at detecting HPV DNA within the saliva. One patient, OPSCC 3, had no detectable HPV DNA in plasma; however, levels in saliva were the highest of the three patients, implying the complementary role that saliva could serve as a source of liquid biopsy. This particular patient had a primary tumor of the tonsil, but limited extent of disease (stage I). Published data suggests that saliva may be more sensitive than plasma for HPV DNA detection in OPSCC [7, 10], but our study suggests that saliva collection and DNA purification techniques can have a dramatic impact on stability of the mononucleosomal-sized cfDNA fraction that may harbor the majority of HPV DNA fragments. It is also possible that the cellular genomic DNA within total salivary DNA preparations could dilute shorter cfDNA constituents and therefore interfere with detectability of HPV DNA. It is possible that our recommended methods and explicit interrogation of shorter salivary DNA fragments could yield greater sensitivity in future studies.

Overall, our study sheds light on a number of important issues for the application of saliva-derived DNA in diagnostics. We have highlighted collection and DNA purification techniques that obtain optimal and consistent yields of mononucleosomal-sized DNA fragments, as well as characterized the different contributing sources within salivary DNA. Expansion of this work in a range of clinical settings will be important to establish the performance characteristics and utility of salivary biomarkers.

## Supporting information

S1 Fig Experimental workflow of healthy and patient samples. Depiction of the techniques used to analyze liquid biopsy-derived DNA.
(TIF)
