# Supplementary figures and images for: Isolation of salivary cell-free DNA for cancer detection
Source: PLoS One. 2023 May 2;18(5):e0285214. doi: 10.1371/journal.pone.0285214 (PMC10153704; doi:10.1371/journal.pone.0285214)

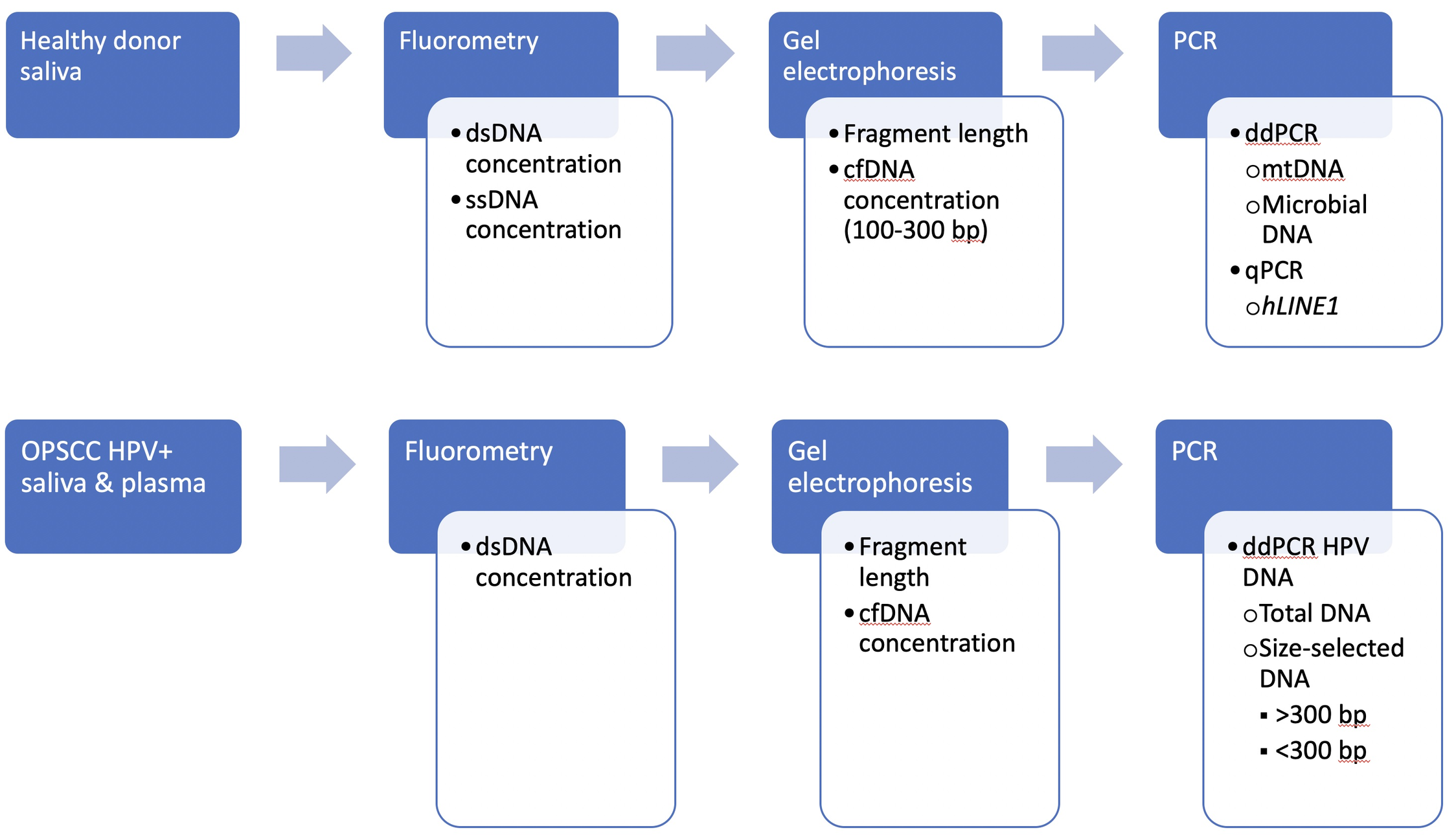

Supplement: S1 Fig — Depiction of the techniques used to analyze liquid biopsy-derived DNA. (TIF) [file pone.0285214.s001.tif]
